# Effects of a postural balance apparatus–assisted combined exercise program on fall-related physical fitness and blood vessel elasticity among older women

**DOI:** 10.1097/MD.0000000000048802

**Published:** 2026-05-15

**Authors:** Jungwoo Lee, Yanggyu Moon, Wi-Young So, Kijeong Kim

**Affiliations:** a School of Exercise & Sport Science, The Balance Korea Company, Ulsan, Republic of Korea; b Department of Sports Medicine, College of Humanities, Korea National University of Transportation, Chungju-si, Republic of Korea; c School of Exercise & Sport Science, University of Ulsan, Ulsan, Republic of Korea.

**Keywords:** blood vessel elasticity, combined exercise, older women, postural balance apparatus

## Abstract

**Background:**

In this study, we evaluated the effects of a combined exercise program incorporating a postural balance apparatus on the fall-related physical fitness and blood vessel elasticity of older women.

**Methods:**

The participants were 36 women aged 65 years or older who did not engage in regular exercise. They were randomly divided into an exercise group (n = 18) and a control group (n = 18). The exercise group underwent a combined exercise program for 12 weeks (60 minutes a day for 5 days a week), whereas the control group engaged in no physical activity or exercise during the 12 weeks. The combined exercise program consisted of aerobic exercise (such as step-ups and the short foot exercise), resistance exercises (such as heel raises, semi-squats, and weight-bearing closed-chain exercises), and joint mobility exercises (such as step-ups with pelvic floor muscle engagement, lower body stretching, and back stretching) to be performed on a postural balance apparatus, which provided an inclined and elastic surface to facilitate ankle dorsiflexion, joint mobility, and proprioceptive stimulation. The exercises were performed at a moderate intensity, maintaining a perceived exertion rating between 11 and 14, for 12 weeks. The outcome variables were measured through experimental procedures. The collected data were analyzed using means, standard deviations, and 2-way repeated measures analysis of variance.

**Results:**

We found a significant improvement in the range of motion for left ankle dorsiflexion in the lying and seated positions, as well as right ankle dorsiflexion in the lying, seated, and prone positions, in the exercise group compared to the control group (*P* < .05). Furthermore, the atherosclerosis left brachial–ankle pulse wave velocity, atherosclerosis right brachial–ankle pulse wave velocity, diastolic blood pressure, and heart rate significantly decreased in the exercise group compared to the control group (*P* < .05).

**Conclusions:**

These results confirmed that the 12-week apparatus-assisted combined exercise program, unlike conventional programs, effectively improves ankle dorsiflexion, vascular elasticity, and cardiovascular health in older women.

## 1. Introduction

Advances in medicine and technology have extended human life expectancy; however, rapid population aging has led to various age-related issues.^[1]^ In 2024, the Republic of Korea became a super-aged society when the proportion of its population aged 65 years or older surpassed 20%.^[2]^ In 2020, the life expectancy of men and women in the Republic of Korea was 80.5 and 86.5 years, respectively, indicating that women live an average of 6 years longer than men. Although the overall average life expectancy in the country is 83.5 years, the healthy life expectancy is 66.3 years. The difference of 17.2 years represents a period of illness, accounting for 20.59% of overall life expectancy.^[3]^ Living in an aging society currently entails facing a prolonged period of illness in later life. According to the 2020 Elderly Status Survey by the Korea Ministry of Health and Welfare, 84.0% of the older population had chronic diseases, 27.8% had multiple chronic conditions, and older individuals had 2.7 chronic diseases on average.^[4]^ Furthermore, medical expenses for individuals aged 65 years or older total USD 31 billion, accounting for 43.4% of the nation’s health insurance costs. Since 2017, this figure has increased by 46% (approximately USD 10 billion), placing a substantial financial burden on the nation’s budget.^[2]^

Aging leads to the degeneration of various organs, with the cardiovascular system deteriorating the fastest.^[5]^ Aging can damage blood vessels and promote fibrosis, leading to reduced arterial elasticity and buffering capacity and increased arterial stiffness.^[6]^ Vascular elasticity decreases with age,^[7]^ while arterial stiffness is inversely related to physical fitness and cardiovascular health.^[8]^ Notably, vascular elasticity serves as a predictive marker for conditions such as hypertension and arteriosclerosis. It is commonly measured using pulse wave velocity (PWV), a key indicator of arterial stiffness.^[9]^ Abnormalities in PWV indicate atherosclerotic vascular damage.^[10]^ In women, circulating estrogen levels considerably decrease because of menopause, which is a natural part of aging.^[11]^ Estrogen plays a key role in regulating blood pressure, and its deficiency can lead to increased blood pressure, a higher heart rate, and greater arterial stiffness.^[12]^ Aging also hampers physical activity and reduces muscle strength, flexibility, and balance, which are essential for maintaining fitness in older adults.^[13]^ Consequently, their walking ability weakens, their posture becomes unstable, and the risk of disabilities and falls increases.^[14]^ According to the 2020 Elderly Status Survey, 41.8% of falls were attributed to physical reasons, and women were more prone to such falls than men.^[15]^ This makes it imperative to address blood vessel elasticity and fall-related physical fitness among older women.

Regular physical activity suppresses the activation of the sympathetic nervous system while strengthening the parasympathetic nervous system, thus improving blood pressure.^[16]^ Regular exercise considerably reduces PWV and decreases arterial stiffness.^[17]^ Additionally, improvements in fitness through training are strongly and independently associated with a reduced risk of cardiovascular diseases.^[18,19]^ Therefore, exercise plays a vital role in the prevention and management of cardiovascular diseases.^[20]^ Notably, cardiorespiratory fitness decreases by 5% to15% every decade after the age of 30 years and is halved by the age of 70 years. However, regular physical activity can reduce this rate by half.^[21]^ Additionally, regular exercise improves balance and mobility, thus reducing the risks of falls, hospitalizations, and death among older adults.^[22]^ It effectively improves frailty, body mass index, muscle mass, muscle strength, walking speed, physical activity levels, and functional abilities.^[23]^ By the age of 80, skeletal mass decreases by an average of 30% to 40% from its peak, particularly in type II muscle fibers.^[24]^ Typically, muscle strength peaks between ages 25 and 35, remains steady until the 40s, and gradually declines thereafter. After the age of 60 years, this decline accelerates, with an average of 4.5% to 5.5% of muscle strength lost every 5 years.^[25]^ Notably, the health status and functional abilities of older adults can vary greatly even at the same age. While some remain healthy, others become pre-frail, frail, or disabled.^[26]^ Therefore, exercise intervention programs should be adjusted according to each person’s fitness level.

Existing strategies for enhancing blood vessel elasticity and fall-related physical fitness in older women involve combined exercise programs using simple tools, such as balls, bands, fans, wooden rods, and chairs.^[27,28]^ Most exercise interventions last 60 to 70 min/session, including warm-up and cool-down periods, which can be lengthy for older individuals. Furthermore, many exercises are performed in seated or lying positions to focus on single-joint movements and ensure joint safety. However, this approach may exacerbate the degeneration of the central nervous system, leading to decreased neural stimulation and physical capabilities. It may increase the risk of musculoskeletal injuries, such as hyperextension and overexertion of joints, muscles, tendons, and ligaments. Furthermore, it may fail to improve dynamic balance and postural control. These limitations emphasize the need for an exercise approach that focuses on multiple-joint movements.

Considering the anatomical, physiological, and environmental factors affecting older women, it is essential to develop a sustainable and practical exercise intervention that can be integrated into daily life and performed without time or space constraints. We developed a combined exercise program that incorporates a postural balance apparatus to facilitate muscle reconstruction, improve joint mobility, and increase older women’s quality of life. Older women participated in the program for 12 weeks (60 min/d, 5 days a week), and we analyzed its impact on fall-related physical fitness and blood vessel elasticity.

## 2. Materials and methods

### 2.1. Participants

Initially, 40 older women (20 per group) were recruited for the study. However, owing to personal circumstances such as illness or travel, several participants with poor adherence were excluded. Consequently, 36 older women completed the intervention and were included in the final analysis.

The eligibility criteria were participants aged 65 years or older, systolic blood pressure below 159 mm Hg and diastolic blood pressure below 99 mm Hg, refraining from regular exercise, and with no neurological or orthopedic diseases. All participants were recruited from the Senior Welfare Center in Ulsan Metropolitan City, Republic of Korea. The sample size was determined through a power analysis conducted using G*Power software (version 3.1.7, Heinrich-Heine-University, Düsseldorf, Germany). Considering a statistical power of 0.80, an α-error probability of 0.05, and an effect size of 0.40, a sample size of 38 was adequate, with a minimum requirement of 16. Considering the characteristics of the target population, a dropout rate exceeding 50% was anticipated.^[29]^

Baseline health status, exercise history, and potential risks were assessed using the Physical Activity Readiness Questionnaire based on the American College of Sports Medicine Guidelines for Exercise Testing and Prescription.^[30]^ After being informed of the study purpose and procedures, participants were randomly assigned to either the exercise (n = 18) or control (n = 18) groups using the sequentially numbered opaque sealed envelopes method. Allocation was concealed using a centralized sequentially numbered opaque sealed envelopes method after participant enrollment, ensuring that investigators could not predict upcoming assignments. In this study, adherence rate was operationally defined as achieving an attendance rate of at least 95% for the intervention sessions. No adverse events occurred during the intervention period. Table 1 presents the physical characteristics of participants in the exercise and control groups. The study protocol was approved by the Institutional Review Board of the University of Ulsan, Ulsan, Republic of Korea (IRB approval number: 2022R0012-003), and all participants provided informed consent prior to enrollment.

**Table 1:** Physical characteristics of participants in the exercise and control groups.

Group	Age (yr)	Height (cm)	Weight (kg)	Skeletal muscle (kg)	Body mass index (kg/m^2^)	Body fat percentage (%)
e.g. (n = 18)	74.21 ± 4.17	158.18 ± 3.95	58.59 ± 5.58	36.06 ± 2.92	23.44 ± 2.09	34.39 ± 3.61
CG (n = 18)	73.07 ± 4.46	153.17 ± 4.49	56.51 ± 8.00	34.13 ± 3.61	24.10 ± 3.43	35.21 ± 7.48

Values are presented as mean ± standard deviation.

CG = control group, EG = exercise group.

### 2.2. Procedure

The exercise program was conducted for 12 weeks (60 min/d, 5 d/wk) in the exercise group, while no physical activities or exercises were undertaken in the control group. All tests for fall-related physical fitness and blood vessel elasticity were conducted twice – once before and once after the intervention – using the same methods at both time points.

### 2.3. Combined exercise program incorporating postural balance apparatus

**Figure 1.**
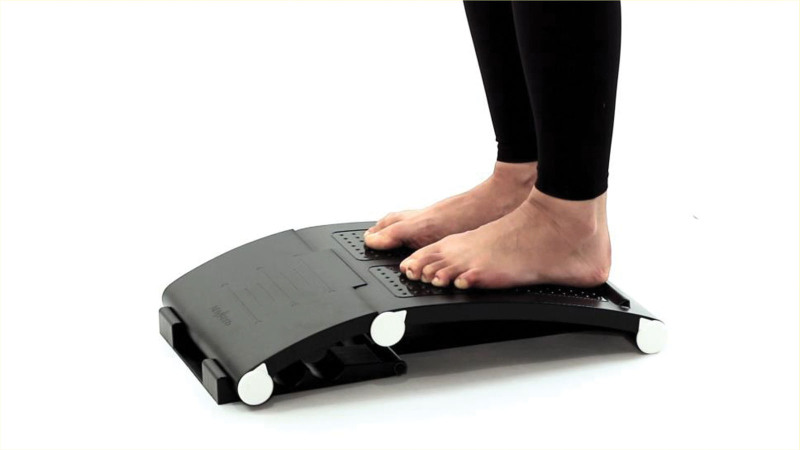
The postural balance apparatus.

The exercise group engaged in a combined exercise program involving the use of a postural balance apparatus (AnyBaro Super, The Balance Korea, Ulsan, Republic of Korea). The postural balance apparatus is shown in Figure 1.

**Figure 2.**
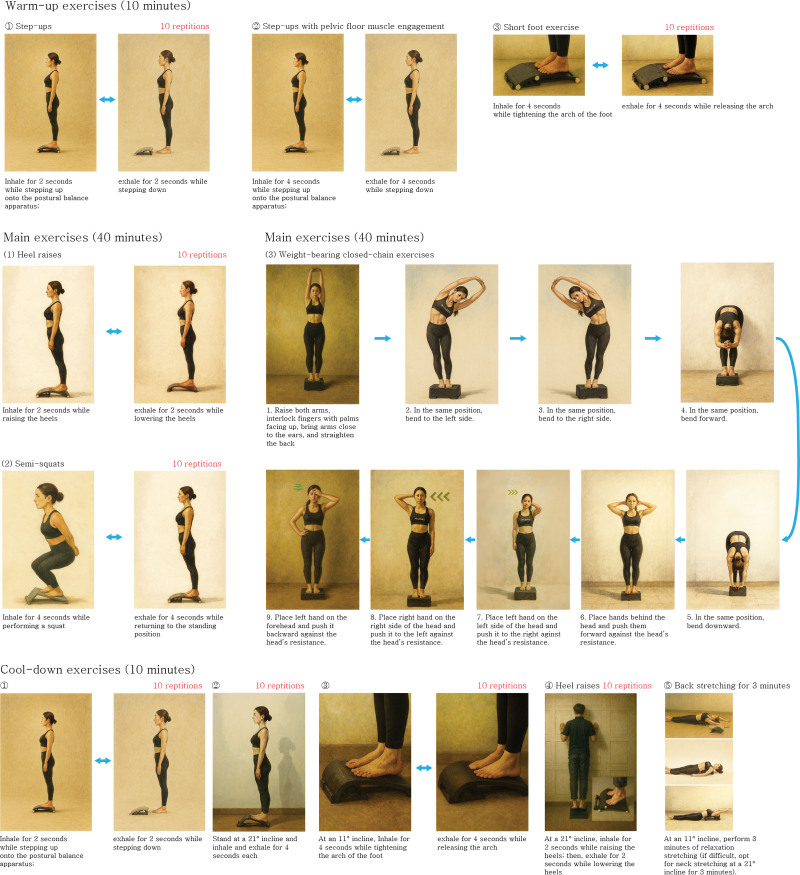
The exercise program with the postural balance apparatus.

Each session consisted of 10 minutes of warm-up exercises, 40 minutes of main exercises, and 10 minutes of cool-down exercises. The main exercises consisted of heel raises, semi-squats, and weight-bearing closed-chain exercises as balance and resistance exercises. The warm-up and cool-down exercises included aerobic exercises, such as step-ups, the short foot exercise, and back stretching exercises, as well as proprioceptive function improvement and flexibility exercises. Fifteen types of exercise interventions listed under the Korea Ministry of Food and Drug Safety’s Class 1 medical device notification (No. 22-716) were reconfigured to be performed on the arch-shaped inclined surface of the postural balance apparatus. We also incorporated closed- and open-chain weight-bearing resistance exercises. The exercise program is described in greater detail in Table 2 and Figure 2. The participants received an exercise program manual that shows how to perform the different exercises with the postural balance apparatus.^[31]^

**Table 2:** Components of the combined exercise program incorporating the use of a postural balance apparatus.

Category	Types of exercise	Exercise content	Number of sets
Warm-up exercises (10 min)	Aerobic exercises and proprioceptive function improvement exercises	(1) Step-ups, 10 repetitions: Inhale for 2 s while stepping up onto the postural balance apparatus; then, exhale for 2 s while stepping down. (2) Step-ups with pelvic floor muscle engagement, 10 repetitions: Inhale for 4 s while stepping up; then, exhale for 4 s while stepping down. (3) Short foot exercise, 10 repetitions: Inhale for 4 s while tightening the arch of the 99foot; then, exhale for 4 s while releasing the arch.	1
Main exercises (40 min)	Balance exercises and resistance exercises	(1) Heel raises, 10 repetitions: Inhale for 2 s while raising the heels; then, exhale for 2 s while lowering the heels. (2) Semi-squats, 10 repetitions: Inhale for 4 s while performing a squat; then, exhale for 4 s while returning to the standing position. (3) Weight-bearing closed-chain exercises: Lower body and pelvic stabilization; inhale and exhale for 4 s each a) Raise both arms, interlock fingers with palms facing up, bring arms close to the ears, and straighten the back. b) In the same position, bend to the left side. c) In the same position, bend to the right side. d) In the same position, bend forward. e) In the same position, bend downward. f) Place hands behind the head and push them forward against the head’s resistance. g) Place left hand on the left side of the head and push it to the right against the head’s resistance. h) Place right hand on the right side of the head and push it to the left against the head’s resistance. i) Place left hand on the forehead and push it backward against the head’s resistance.	5
Cool-down exercises (10 min)	Aerobic exercises, proprioceptive function improvement exercises, and flexibility exercises	(1) Step-ups, 10 repetitions: Inhale for 2 s while stepping up onto the postural balance apparatus; then, exhale for 2 s while stepping down. (2) Lower body stretching, 10 repetitions: Stand at a 21° incline and inhale and exhale for 4 s each. (3) Short foot exercise, 10 repetitions: At an 11° incline, inhale for 4 s while tightening the arch; then, exhale for 4 s while releasing the arch. (4) Heel raises, 10 repetitions: At a 21° incline, inhale for 2 s while raising the heels; then, exhale for 2 s while lowering the heels. (5) Back stretching for 3 min: At an 11° incline, perform 3 min of relaxation stretching (if difficult, opt for neck stretching at a 21° incline for 3 min)	1

During the adaptation phase (weeks 1–4), exercises were conducted on an 11° inclined plane. In the functional improvement phase (weeks 5–8), the incline was increased to 21°. Subsequently, in the functional enhancement phase (weeks 9–12), the incline was further increased to 25°, depending on the participant’s muscle strength and exercise proficiency. Additionally, the exercise intensity was adjusted based on the participants’ perceived exertion levels, which were measured using the 20-point rating of perceived exertion scale.^[32]^ During the adaptation phase, the rating of perceived exertion was maintained between 10 (“very easy”) and 12 (“fairly easy”). Participants’ perceived exertion level was observed during the exercises, and the program was adjusted accordingly. After the adaptation phase, the perceived exertion level was moderately increased, maintaining a rating of perceived exertion score between 11 (“fairly easy”) and 14 (“somewhat hard”). The participants’ exertion levels were determined once a week throughout the program.

Before the exercise program, a 1-hour instructional session was conducted in the fitness training room of the Senior Welfare Center in Ulsan Metropolitan City, Republic of Korea. In this session, a professional instructor discussed muscle movements, proper posture alignment, exercise intensity, and safe use of the exercise apparatus. Subsequently, the participants were provided with a posture balance apparatus and an exercise program video that included movement explanations. During the intervention, the exercise group engaged in 60 minutes of daily exercise by following the exercise program video. In the adaptation phase, exercises were conducted with support from either a wall or chair to ensure safety. Monitoring sessions were held for 60 minutes each week at the fitness training room of the Senior Welfare Center to assess the performance of the exercise group.

### 2.4. Measurements

#### 2.4.1. Fall-related physical fitness

To assess fall-related physical fitness, we used variables from the Senior Fitness Test, which was developed in 2012 and is currently utilized in the Korea National Fitness 100 program for older individuals.^[33]^ We also used the fall-related physical fitness variables from Rikli and Jones’ Senior Fitness Test protocol.^[34]^ All variables were modified to fit the context of this study.

##### 2.4.1.1. Body composition

We measured the participants’ height, weight, muscle mass, body water, protein levels, minerals, body fat, skeletal muscle mass, body fat mass, and body fat percentage using a bioelectrical impedance analyzer (Inbody 370, Biospace, Republic of Korea). After removing all metal objects, the participants, dressed in light clothes and barefoot, were instructed to step onto the device for measurement.

##### 2.4.1.2. Joint range of motion (ankle dorsiflexion)

We measured the active range of motion for ankle dorsiflexion in lying, seated, and prone positions using a plastic goniometer. The central axis of the goniometer was positioned at the lateral malleolus of the fibula, with the head of the fibula serving as the reference point. The proximal arm of the goniometer was aligned with the central line along the lateral surface of the calf, while the distal arm was positioned parallel to the lateral surface of the fifth metatarsal bone. The measurements were obtained with the plantar as the axis.^[35]^ Care was taken to maintain a 90° knee bend while preventing foot rotation or inward/outward spreading in the seated and prone positions. Each measurement posture was maintained for 5 seconds, with a 3-minute rest period between posture changes.

##### 2.4.1.3. Flexibility

The participants’ flexibility was determined based on the seated forward bend posture. The participants were asked to sit on a chair with their knees extended and reach forward with their hands overlapping. We measured the distance between the fingertips and the toes.

##### 2.4.1.4. Balance

Balance was assessed using the Single-Leg Standing with Eyes Open test. The participants were instructed to stand on 1 leg. The stopwatch was started upon the tester’s signal to begin, and measurements were taken separately for the open- and closed-eye conditions. This test was conducted separately for each leg.

##### 2.4.1.5. Upper body strength

The participants’ upper body strength was determined based on their grip strength, which was measured using a grip strength meter (GRIP-D 5101; Takei, Co., Japan). The participants alternated hands, starting with the dominant hand, and the highest recorded value was used as the final value.

##### 2.4.1.6. Lower body endurance

The participants’ lower body endurance was measured using the 30-second sit-to-stand test. We recorded the number of times the participant could stand up from a seated position on a chair and sit back down within 30 seconds.

##### 2.4.1.7. Cardiorespiratory endurance

Cardiorespiratory endurance was measured using the 2-minute on-the-spot walking exercise. We asked the participant to walk in place for 2 minutes and recorded the number of times the knee reached the midpoint between the patella and the iliac crest.

#### 2.4.2. Blood vessel elasticity

Blood vessel elasticity was assessed using the brachial–ankle PWV (baPWV), ankle–brachial index, systolic blood pressure, diastolic blood pressure, and heart rate. Before the measurements, the participants were asked to rest in a supine position for more than 10 minutes. Measurements were subsequently performed using an oscillometric blood pressure waveform analyzer (HBP-8000, Omron Healthcare Co., Kyoto, Japan). This device allows automated rapid exhaust and pressurization through electronic control valves, with decompression also being automated. Pressure was detected using a semiconductor piezoelectric sensor. The accuracy of blood pressure measurements was maintained within an average deviation of ±5 mm Hg and a standard deviation of 8 mm Hg compared with manual auscultation by trained professionals. Pulse measurements were accurate to ±2% or ±2 beats, whichever was greater. Furthermore, the PWV and ankle–brachial index were calculated in the following manner.

PWV = L/PTT, where L denotes the distance the pulse travels and PTT signifies the time taken for the pulse wave to travel.

Ankle–brachial index = ankle systolic blood pressure/higher of left or right arm systolic blood pressure

### 2.5. Data analysis

Means and standard deviations for fall-related physical fitness and blood vessel elasticity variables were calculated for both groups. Two-way repeated measures analysis of variance was conducted to assess mean differences between pre- and post-intervention measurements and to analyze interactions between the groups. Statistical assumptions, such as normality and homogeneity of variances, were assessed prior to the analysis. No adjustments for multiple comparisons were made in the statistical analyses. Differences were considered statistically significant at α = 0.05. All statistical analyses were performed using SPSS Statistics version 22.0 (IBM Corp., Armonk).

## 3. Results

As shown in Table 3, a significant interaction between time and group was found for the lying position left ankle dorsiflexion, lying position right ankle dorsiflexion, seated position left ankle dorsiflexion, seated position right ankle dorsiflexion, and prone position right ankle dorsiflexion range of motion (*P* < .05).

**Table 3:** Descriptive statistics and analysis of variance results for joint range of motion.

Variable	Group	Pre-intervention	Post-intervention	95% CI (EMMs)	Interaction	*F*	*P*
Lying position left ankle dorsiflexion	e.g. (n = 18)	10.19 ± 4.45	14.47 ± 3.63	12.44–15.89	Time × group	16.729	<.001***
	CG (n = 18)	8.23 ± 2.71	8.92 ± 2.77	7.51–10.96			
Lying position right ankle dorsiflexion	e.g. (n = 18)	10.25 ± 3.86	14.34 ± 3.54	12.46–15.81	Time × group	17.647	<.001***
	CG (n = 18)	8.71 ± 2.69	9.04 ± 2.52	7.57–10.92			
Seated position left ankle dorsiflexion	e.g. (n = 18)	10.17 ± 3.21	13.67 ± 3.33	11.86–15.10	Time × group	15.601	.001**
	CG (n = 18)	9.05 ± 3.18	8.85 ± 2.77	7.43–10.67			
Seated position right ankle dorsiflexion	e.g. (n = 18)	10.49 ± 2.93	14.32 ± 3.31	12.58–15.72	Time × group	21.545	<.001***
	CG (n = 18)	9.63 ± 3.16	8.94 ± 2.76	7.55–10.69			
Prone position left ankle dorsiflexion	e.g. (n = 18)	15.00 ± 3.24	17.82 ± 3.94	14.91–19.23	Time × group	3.846	.061
	CG (n = 18)	10.39 ± 3.58	13.14 ± 3.28	11.73–16.05			
Prone position right ankle dorsiflexion	e.g. (n = 18)	15.74 ± 3.33	18.29 ± 3.80	15.55–19.62	Time × group	5.836	.023*
	CG (n = 18)	11.19 ± 3.35	13.19 ± 2.94	11.86–15.92			

Values are presented as mean ± standard deviation.

CG = control group, CI = confidence interval, EG = exercise group, EMM = estimated marginal mean.

* *P* < .05, tested using 2-way repeated measures analysis of variance.

** *P* < .01, tested using 2-way repeated measures analysis of variance.

*** *P* < .001, tested using 2-way repeated measures analysis of variance.

These findings suggest that the intervention was effective in improving joint range of motion. However, we found no significant interaction between time and group for flexibility, left balance, right balance, left upper body strength, right upper body strength, lower body endurance, and cardiorespiratory endurance (Table 4; *P* > .05).

**Table 4:** Descriptive statistics and analysis of variance results for other fall-related physical fitness variables.

Variable	Group	Pre-intervention	Post-intervention	95% CI (EMMs)	Interaction	*F*	*P*
Flexibility (cm)	e.g. (n = 18)	6.68 ± 7.38	12.00 ± 9.02	9.28–16.09	Time × group	0.215	.647
	CG (n = 18)	8.82 ± 9.22	12.29 ± 7.01	8.19–15.00			
Balance left (s)	e.g. (n = 18)	17.26 ± 19.29	34.17 ± 47.59	15.05–56.22	Time × group	0.346	.562
	CG (n = 18)	20.40 ± 22.44	28.77 ± 34.30	6.73–47.89			
Balance right (s)	e.g. (n = 18)	23.11 ± 21.63	30.11 ± 33.10	14.96–40.07	Time × group	0.172	.681
	CG (n = 18)	17.51 ± 15.90	21.31 ± 22.67	11.36–36.47			
Upper body strength left (kg)	e.g. (n = 18)	21.89 ± 3.23	21.85 ± 2.67	20.20–22.72	Time × group	0.010	.920
	CG (n = 18)	20.85 ± 4.67	20.99 ± 4.58	20.11–22.64			
Upper body strength right (kg)	e.g. (n = 18)	22.58 ± 3.18	22.96 ± 2.78	20.89–23.65	Time × group	0.001	.981
	CG (n = 18)	20.41 ± 2.54	22.28 ± 4.32	20.81–23.69			
Lower body endurance (reps/30 s)	e.g. (n = 18)	13.00 ± 3.51	13.57 ± 2.44	12.26–14.75	Time × group	0.163	.690
	CG (n = 18)	12.71 ± 3.29	13.79 ± 2.94	12.61–15.09			
Cardiorespiratory endurance (reps/2 min)	e.g. (n = 18)	96.50 ± 17.65	109.79 ± 14.35	101.43–119.45	Time × group	1.581	.220
	CG (n = 18)	100.50 ± 19.23	103.29 ± 19.48	93.62–111.65			

Values are presented as mean ± standard deviation.

CG = control group, CI = confidence interval, e.g. = exercise group, EMM = estimated marginal mean.

* *P* < .05, tested using 2-way repeated measures analysis of variance.

** *P* < .01, tested using 2-way repeated measures analysis of variance.

*** *P* < .001, tested using 2-way repeated measures analysis of variance.

Regarding blood vessel elasticity, we found a significant interaction between time and group for left baPWV, right baPWV, diastolic blood pressure, and heart rate (Table 5; *P* < .05).

**Table 5:** Descriptive statistics and analysis of variance results for blood vessel elasticity variables.

Variable	Group	Pre-intervention	Post-intervention	95% CI (EMMs)	Interaction	*F*	*P*
Left baPWV (cm/s)	e.g. (n = 18)	1706.14 ± 273.21	1597.50 ± 232.27	1471.49–1646.52	Time × group	9.809	.004**
	CG (n = 18)	1620.50 ± 215.07	1710.21 ± 302.64	1661.20–1836.22			
Right baPWV (cm/s)	e.g. (n = 18)	1736.07 ± 293.55	1632.29 ± 252.17	1495.37–1668.91	Time × group	9.601	.005**
	CG (n = 18)	1616.86 ± 211.06	1719.21 ± 274.50	1682.59–1856.13			
Left ABI (points)	e.g. (n = 18)	1.15 ± 0.08	1.18 ± 0.04	1.15–1.22	Time × group	0.229	.636
	CG (n = 18)	1.19 ± 0.07	1.17 ± 0.09	1.14–1.21			
Right ABI (points)	e.g. (n = 18)	1.16 ± 0.07	1.19 ± 0.06	1.16–1.22	Time × group	0.455	.506
	CG (n = 18)	1.18 ± 0.07	1.18 ± 0.08	1.14–1.21			
SBP (mm Hg)	e.g. (n = 18)	135.36 ± 15.54	127.64 ± 5.17	120.76–132.63	Time × group	1.006	.326
	CG (n = 18)	130.14 ± 15.27	129.86 ± 16.02	124.87–136.74			
DBP (mm Hg)	e.g. (n = 18)	68.21 ± 10.12	62.07 ± 4.46	57.52–66.49	Time × group	5.692	.025*
	CG (n = 18)	67.93 ± 8.98	69.29 ± 12.17	64.87–73.84			
Heart rate (bpm)	e.g. (n = 18)	75.71 ± 9.08	70.93 ± 5.94	68.66–75.12	Time × group	6.907	.014*
	CG (n = 18)	78.86 ± 11.38	78.71 ± 10.47	74.52–80.98			

Values are presented as mean ± standard deviation.

ABI = ankle–brachial index, baPWV = brachial–ankle pulse wave velocity, CI = confidence interval, CG = control group, DBP = diastolic blood pressure, e.g. = exercise group, EMM = estimated marginal mean, SBP = systolic blood pressure.

* *P* < .05, tested using 2-way repeated measures analysis of variance.

** *P* < .01, tested using 2-way repeated measures analysis of variance.

## 4. Discussion

This study is significant because it involved older women (average age over 70 years) participating in a structured exercise program for 60 minutes a day, 5 days a week, over a 12-week period, guided by workout videos and exercise-intensity checklists. The incorporation of a postural balance apparatus in the combined exercise program yielded notable results among these women. In particular, the postural balance apparatus–assisted combined exercise program led to reductions in PWV and diastolic blood pressure, along with improvements in arterial stenosis and resting heart rate. These findings suggest that the postural balance apparatus–assisted combined exercise program can induce positive changes in cardiovascular health among older women. Additionally, it may motivate them to adopt a proactive approach to self-managed healthcare.

Optimal musculoskeletal flexibility can enhance the range of motion of joints and reduce the risk of musculoskeletal injuries, thereby improving exercise performance.^[33-35]^ Resistance exercises can be performed using one’s body weight or weight machines, offering a versatile range of exercise options. These exercises are broadly categorized into open- and closed-chain exercises based on how the limbs interact with the environment during the exercise.^[36]^ In closed-chain exercises, the distal parts of the limbs remain fixed, and resistance is applied simultaneously to the proximal and distal parts.^[37]^ In contrast, open-chain exercises involve the free movement of the distal parts, while the proximal part remains fixed. Because of the unpredictability of movements in other parts of the chain in open-chain exercises, such as those in free space, ensuring the biomechanical stability of the joints is challenging.^[38]^

To avoid excessive load on the vulnerable joints of older adults, exercises should be conducted within a limited range of motion and under careful observation of professionals. Closed-chain exercises are characterized by increased joint compression, improved joint alignment and stability, reduced shear forces and acceleration, greater resistance, enhanced proprioceptor activation, and dynamic stability. These exercises produce less biomechanical stress and force that could threaten healing structures, making them safer than open-chain exercises from a biomechanical perspective.^[39]^ In closed-chain exercises, the cooperative contraction of multiple muscles results in complex muscle actions that enhance joint stability. This makes closed-chain exercises particularly beneficial in early rehabilitation, where joint stability is crucial.^[40]^ The intervention of weight-bearing exercises for older women suggests the need for closed-chain exercises that incorporate gradual weight-bearing resistance. This approach should consider individual physical characteristics and exercise capabilities, ensuring joint stability within a limited range of motion.

The reaction time of ankle muscles for maintaining balance is markedly longer in older adults, and ankle muscle strength decreases significantly with age. Specifically, weakened dorsiflexor muscles of the foot markedly reduce the balance maintenance capabilities of older adults.^[41]^ In this study, the postural balance apparatus had an arch-shaped inclined surface made of elastic materials, ideal for rebuilding muscles in older individuals and suitable for joint mobility training. This design allowed the performance of various foot-strengthening exercises without directly affecting the joints, thereby reducing exercise fatigue. A previous study revealed that, similar to the effects of spinal manual therapy and other physical activities, standing on a 16° inclined surface reduces overall lower back pain by 59.4%.^[42]^ However, other studies have cautioned that increasing surface inclination to 15° may lead to excessive dorsiflexion, adversely affecting the knee and ankle joints. They have also suggested that an incline between 10° and 16° is recommended for standing interventions aimed at enhancing walking activities.^[43,44]^ In this study, the postural balance apparatus could be manually adjusted to 11°, 21°, and 25°. This allowed older women to easily modify exercise intensity according to their physical characteristics and proficiency, leading to enhanced exercise performance. Its arch-shaped design ensured the smooth maintenance of the body’s natural curves. Consequently, it facilitated not only stretching and weight-bearing exercises but also exercises strengthening the intrinsic foot muscles without stimulating the neck, lower back, spinal joints, or foot arches.

The results of this study revealed that using a postural balance apparatus in a combined exercise program improves the ankle dorsiflexion range of motion in various positions. Humans have adapted to an upright posture by positioning the calcaneus beneath the talus and allowing the gastrocnemius–soleus–Achilles tendon complex to relax passively; in this way, the ankle achieves dorsiflexion of 90°.^[45]^ This mechanism plays a crucial role in weight-bearing and walking activities. In an upright posture, balance is maintained through the controlled elongation and contraction of the anterior and posterior muscle complexes below the knee joint. During walking, this posture facilitates natural ankle dorsiflexion at heel strike and helps balance ground reaction forces during terminal stance and toe-off.^[46]^ The connection between the gastrocnemius–soleus–Achilles tendon complex and the plantar muscle complex via the posterior protuberance of the calcaneus helps reduce the overload from the lower leg to the toes during weight-bearing and walking, thus allowing efficiency maximization.^[47]^ Tension and tightening in the gastrocnemius–soleus–Achilles tendon complex can cause disharmony in the movement of the ankle joint and the entire foot during weight-bearing and walking, possibly causing an unbalanced force distribution.^[48]^ Therefore, a postural balance apparatus can be used while performing combined exercises to effectively treat important muscles and joints involved in weight-bearing and walking.

Appropriate physical activity and regular exercise have numerous benefits, including improvements in cardiopulmonary function, blood pressure, muscle strength, joint flexibility, and physical mobility.^[49-51]^ Long-term regular exercise reduces the thickness of the carotid artery and increases its lumen diameter, potentially decreasing PWV and preventing atherosclerosis-induced cardiovascular and cerebrovascular diseases.^[17]^ Pinckard et al^[52]^ reported that regular exercise lowers the resting heart rate, increases resistance to arrhythmias in the myocardium, improves blood lipids, and reduces arteriosclerosis. Yoshizawa et al^[53]^ found that moderate-intensity resistance exercise decreases the carotid–femoral artery PWV. Additionally, resistance exercises enhance quality of life and reduce or prevent the risk of cardiovascular diseases.^[34]^ Furthermore, previous studies have revealed that blood pressure reduction is closely associated with the restoration of blood vessel elasticity achieved by decreasing arterial fatigue and degenerative sclerosis.^[54-56]^ Lo et al^[57]^ supported these findings and reported that an 8-week Tai Chi exercise program improves exercise behavior and reduces blood pressure in patients with hypertension.

The findings of this study revealed that our 12-week postural balance apparatus-assisted combined exercise program improves diastolic blood pressure, heart rate, and arterial stenosis on both sides among older women. These findings align with those of previous studies demonstrating that diversifying exercises (such as aerobic, combined, and resistance exercises) improves aortic elasticity, reduces peripheral vascular resistance, restores vascular elasticity, changes blood volume, and promotes positive shifts in the endocrine system.^[58,59]^ Moreover, standing on the inclined surface of a postural balance apparatus and performing combined exercises mostly involves ankle dorsiflexion while stabilizing the lower body. In this position, the torso and upper body move, aligning the muscles and joints of the entire body. This alignment provides continuous mechanical stimulation to the gastrocnemius muscle, which occupies approximately two-thirds of the calf’s posterior. In turn, the mechanical stimulation increases blood flow to the skeletal muscles, increasing vascular pressure, expanding blood vessels, and activating the sympathetic nervous system, which positively affects vascular elasticity.^[60,61]^ In addition, training-induced neural adaptations may positively influence autonomic regulation, resulting in reduced resting heart rate, diastolic blood pressure, and mechanical stress on the vascular walls. Repeated contractions of the lower limb musculature can further enhance venous return and peripheral circulation, contributing to improved vascular function. Furthermore, weight-bearing dorsiflexion exercises on an inclined surface may provide mechanical stimulation to the vascular endothelium, increasing flow-mediated dilation and ultimately enhancing vascular elasticity. Therefore, participating in a postural balance apparatus–assisted combined exercise program for 60 minutes a day can bolster blood vessel elasticity and fall-related physical fitness among older women. Moreover, it can reduce the risk of cardiovascular diseases and falls among these individuals and help them age healthily and free from morbidity.

This study has several limitations. First, although the 12-week duration and small number of participants showed significant positive outcomes, involving a larger group over a longer period, such as 5 months or more, may yield more substantial results. Additionally, clinical reference values and long-term follow-up assessments should be incorporated in future studies for a more accurate insight into the clinical significance of the findings. Second, the participants were limited to older women, which prevented sex-based comparisons. Thus, future studies should include both male and female participants. Third, although the participants were instructed to maintain their usual lifestyle patterns and refrain from engaging in additional physical activities outside of the exercise program during the intervention period, we did not assess potential confounding factors, such as baseline physical activity levels and dietary habits. In future studies, the researchers should quantitatively assess and control for these variables. Fourth, it is possible that the uniform exercise program overlooked individual fitness levels, risking overload or insufficient stimulus. Fifth, crucial parameters such as upper limb strength (e.g., grip strength, arm resistance exercises), core stability, and functional activities of daily living were not evaluated, which narrowed the scope of the study. These additions will provide a more comprehensive picture of the intervention’s effectiveness. Sixth, although the appropriate sample size was determined through a power analysis conducted using the G* Power software, the sample size was relatively small, which restricts generalizability. Further large-sample studies are necessary. Seventh, assumptions of normality and homogeneity in this study were checked. However, adjustments for multiple comparisons were not applied. Finally, because this study was conducted among older women who resided in a specific region, the findings may not be generalizable to all older women in the Republic of Korea. Expanding the study’s geographic area, such as to the entire Ulsan region, may enhance the reliability of the findings. Considering these aspects in future research may improve the diversity and effectiveness of welfare programs for older individuals.

## 5. Conclusion

A 12-week postural balance apparatus–assisted combined exercise program can effectively improve ankle dorsiflexion, baPWV, diastolic blood pressure, and resting heart rate among women in their 70s. Future research should evaluate the effects of different exercise parameters, such as duration (e.g., short-term vs long-term), intensity (e.g., moderate vs high), and frequency. Additionally, including a larger and more diverse sample (varying age groups and sexes), clinical outcome measures, and long-term follow-ups will further validate the findings. These efforts are expected to help develop more individualized and effective exercise programs, promoting healthier and disease-free life expectancy in the aging population.

## Author contributions

**Conceptualization:** Jungwoo Lee, Kijeong Kim.

**Data curation:** Jungwoo Lee, Kijeong Kim.

**Formal analysis:** Jungwoo Lee, Kijeong Kim.

**Funding acquisition:** Jungwoo Lee, Kijeong Kim.

**Investigation:** Jungwoo Lee, Yanggyu Moon, Wi-Young So.

**Methodology:** Yanggyu Moon, Wi-Young So.

**Project administration:** Yanggyu Moon, Wi-Young So.

**Resources:** Yanggyu Moon, Wi-Young So, Kijeong Kim.

**Software:** Yanggyu Moon, Wi-Young So, Kijeong Kim.

**Supervision:** Yanggyu Moon, Wi-Young So, Kijeong Kim.

**Validation:** Yanggyu Moon, Wi-Young So, Kijeong Kim.

**Visualization:** Yanggyu Moon, Wi-Young So, Kijeong Kim.

**Writing** – **original draft:** Jungwoo Lee, Yanggyu Moon.

**Writing** – **review & editing:** Wi-Young So, Kijeong Kim.

## References

[1] Yesilbalkan OU Karadahovan A. The frequency of falls in elderly individuals living in Narlidere rest home and the evaluation of the affection factors. Truk J Geriatri. 2005;8:72–7.

[2] Korea Health Insurance Review and Assessment Service, National Health Insurance Corporation. 2022 National Health Insurance Statistical Yearbook. Korea Health Insurance Review and Assessment Service, National Health Insurance Corporation; 2022. https://www.hira.or.kr/bbsDummy.do?pgmid=HIRAJ030000007001&brdScnBltNo=4&brdBltNo=7. Accessed January 29, 2025.

[3] Statistics Korea. Social Indicators in Korea 2021. Seoul, Statistics Korea; 2022.

[4] Korea Ministry of Health and Welfare. 2020 Survey of Seniors. Korea Ministry of Health and Welfare; 2021. https://www.mohw.go.kr/board.es?mid=a10411010200&bid=0019&act=view&list_no=366496

[5] Evans EM Racette SB Peterson LR Villareal DT Greiwe JS Holloszy JO. Aerobic power and insulin action improve in response to endurance exercise training in healthy 77–87 yr olds. J Appl Physiol. 2005;98:40–5. 15591302 10.1152/japplphysiol.00928.2004

[6] Braith RW Beck DT. Resistance exercise: training adaptations and developing a safe exercise prescription. Heart Fail Rev. 2008;13:69–79. 17932746 10.1007/s10741-007-9055-9

[7] Lakatta EG Levy D. Arterial and cardiac aging: major shareholders in cardiovascular disease enterprises: Part I: aging arteries: a “set up” for vascular disease. Circulation. 2003;107:139–46. 12515756 10.1161/01.cir.0000048892.83521.58

[8] Chen Y Chandler MP DiCarlo SE. Acute exercise attenuates cardiac autonomic regulation in hypertensive rats. Hypertension. 1995;26:676–83. 7558230 10.1161/01.hyp.26.4.676

[9] O’Neal DN Dragicevic G Rowley KG. A cross-sectional study of the effects of type 2 diabetes and other cardiovascular risk factors on structure and function of nonstenotic arteries of the lower limb. Diabetes Care. 2003;26:199–205. 12502681 10.2337/diacare.26.1.199

[10] Yamashina A Tomiyama H Takeda K. Validity, reproducibility, and clinical significance of noninvasive brachial-ankle pulse wave velocity measurement. Hypertens Res. 2002;25:359–64. 12135313 10.1291/hypres.25.359

[11] Baker C Benayoun BA. Menopause is more than just loss of fertility. Public Policy Aging Rep.. 2023;33:113–9. 38155935 10.1093/ppar/prad023 PMC10751372

[12] Suzuki H Kondo K. Pulse wave velocity in postmenopausal women. Pulse (Basel). 2013;1:4–13. 26587424 10.1159/000348416 PMC4315339

[13] Gauchard GC Gangloff P Jeandel C Perrin PP. Physical activity improves gaze and posture control in the elderly. Neurosci Res. 2003;45:409–17. 12657454 10.1016/s0168-0102(03)00008-7

[14] Lehtola S Hannine L Paatalo M. The incidence of falls during a six-month exercise trial and four-month follow up among home dwelling persons aged 70-75 years. Liikunta Tied. 2000;6:41–7.

[15] Lee YK Kim SJ Hwang NH. 2020 Survey of Seniors. Ministry of Health and Welfare, Korea Institute of Health and Social Affairs; 2020.

[16] Duncan JJ Farr JE Upton SJ Hagan RD Oglesby ME Blair SN. The effects of aerobic exercise on plasma catecholamines and blood pressure in patients with mild essential hypertension. JAMA. 1985;254:2609–13. 4057469

[17] Kadoglou NP Iliadis F Liapis CD. Exercise and carotid atherosclerosis. Eur J Vasc Endovasc Surg. 2008;35:264–72. 17988901 10.1016/j.ejvs.2007.08.022

[18] Blair SN Kampert JB Kohl HW 3rd. Influences of cardiorespiratory fitness and other precursors on cardiovascular disease and all-cause mortality in men and women. JAMA. 1996;276:205–10. 8667564

[19] Gulati M Pandey DK Arnsdorf MF. Exercise capacity and the risk of death in women: the St James women take heart project. Circulation. 2003;108:1554–9. 12975254 10.1161/01.CIR.0000091080.57509.E9

[20] Colberg SR Albright AL Blissmer BJ. Exercise and type 2 diabetes: American College of Sports Medicine and the American Diabetes Association: joint position statement. Exercise and type 2 diabetes. Med Sci Sports Exerc. 2010;42:2282–303. 21084931 10.1249/MSS.0b013e3181eeb61c

[21] Paterson DH Warburton DE. Physical activity and functional limitations in older adults: a systematic review related to Canada’s physical activity guidelines. Int J Behav Nutr Phys Act. 2010;7:38. 20459782 10.1186/1479-5868-7-38 PMC2882898

[22] Giné-Garriga M Guerra M Pagès E Manini TM Jiménez R Unnithan VB. The effect of functional circuit training on physical frailty in frail older adults: a randomized controlled trial. J Aging Phys Act. 2010;18:401–24. 20956842 10.1123/japa.18.4.401

[23] Dedeyne L Deschodt M Verschueren S Tournoy J Gielen E. Effects of multi-domain interventions in (pre)frail elderly on frailty, functional, and cognitive status: a systematic review. Clin Interv Aging. 2017;12:873–96. 28579766 10.2147/CIA.S130794 PMC5448695

[24] Walston JD. Sarcopenia in older adults. Curr Opin Rheumatol. 2012;24:623–7. 22955023 10.1097/BOR.0b013e328358d59b PMC4066461

[25] Pedrero-Chamizo R Gómez-Cabello A Delgado S. Physical fitness levels among independent non-institutionalized Spanish elderly: the elderly EXERNET multi-center study. Arch Gerontol Geriatr. 2012;55:406–16. 22424779 10.1016/j.archger.2012.02.004

[26] Mitnitski AB Graham JE Mogilner AJ Rockwood K. Frailty, fitness and late-life mortality in relation to chronological and biological age. BMC Geriatr. 2002;2:1. 11897015 10.1186/1471-2318-2-1 PMC88955

[27] Jung C Park WY. Effects of complex exercise with tubing and swiss ball on farmer’s syndrome and fall related fitness variables in elderly woman. KSSLS. 2017;70:573–82.

[28] Son NJ Yi KO An JY. The effect of exercise program for prevention of falling on physical fitness, posture and fall prevention self-efficacy for elderly women. J Korea Gerontol Soc. 2017;37:237–50.

[29] Faul F Erdfelder E Lang A-G Buchner A. G*Power 3: a flexible statistical power analysis program for the social, behavioral, and biomedical sciences. Behav Res Methods. 2007;39:175–91. 17695343 10.3758/bf03193146

[30] American College of Sports Medicine. ACSM’s guidelines for exercise testing and prescription. Eleventh Edition. Wolters Kluwer; 2023. 10.1249/JSR.0b013e31829a68cf 23851406

[31] Tufail M Lee H Moon Y Kim H Kim K. Interdisciplinary co-design research practice in the rehabilitation of elderly individuals with chronic low back pain from a senior care center in South Korea. Appl Sci. 2022;12:4687.

[32] Borg G. Borg’s Perceived Exertion And Pain Scales. Human Kinetics; 1998:104.

[33] Park SJ Song HS Kim GJ. Effects of Korean national fitness award program group exercise on daily fitness and balance confidence among the elderly participants. Korean J Sport Sci. 2014;25:650663.

[34] Rikli RE Jones CJ. Senior Fitness Test Manual. 2nd edition. Human Kinetics; 2013.

[35] Cynthia CN Joyce WD. Measurement of Joint Motion: A Guide to Goniometry, 5E. The F. A. Davis Company; 2016: 387–418.

[36] Williams MA Haskell WL Ades PA. Resistance exercise in individuals with and without cardiovascular disease: 2007 update: a scientific statement from the American heart association council on clinical cardiology and council on nutrition, physical activity, and metabolism. Circulation. 2007;116:572–84. 17638929 10.1161/CIRCULATIONAHA.107.185214

[37] Prentice WE Voight MI. Techniques in musculoskeletal rehabilitation. Williams & Wilkins, Baltimore; 1999.

[38] Karandikar N Vargas OO. Kinetic chains: a review of the concept and its clinical application. PM R. 2011;3:739–45. 21871418 10.1016/j.pmrj.2011.02.021

[39] Butler DL Noyes FR Grood ES. Ligamentous restraints to anterior-posterior drawer in the human knee. A biomechanical study. J Bone Joint Surg Am. 1980;62:259–70. 7358757

[40] Heller BM Pincivero DM. The effects of ACL injury on lower extremity activation during closed kinetic chain exercise. J Sports Med Phys Fitness. 2003;43:180–8. 12853899

[41] Shellbourne D Nitz P. Accelerated rehabilitation after anterior cruciate ligament reconstruction. Am J Sports Med. 1990;18:292–9. 2372081 10.1177/036354659001800313

[42] Keshner EA Allum JH Honegger F. Predictors of less stable postural responses to support surface rotations in healthy human elderly. J Vestib Res. 1993;3:419–29. 8275275

[43] Nelson-Wong E Callaghan JP. The impact of a sloped surface on low back pain during prolonged standing work: a biomechanical analysis. Appl Ergon. 2010;41:787–95. 20116046 10.1016/j.apergo.2010.01.005

[44] Mezzarane RA Kohn AF. Control of upright stance over inclined surfaces. Exp Brain Res. 2007;180:377–88. 17279384 10.1007/s00221-007-0865-8

[45] Vieira L. Phylogenetics of the fascial system. Cureus. 2020;12:e10787. 33154854 10.7759/cureus.10787 PMC7606207

[46] Chun DI Won SH. Does achilles tendon shortening mean pathologic lesions? J Korean Foot Ankle Soc. 2021;25:55–60.

[47] Zwirner J Zhang M Ondruschka B Akita K Hammer N. An ossifying bridge - on the structural continuity between the Achilles tendon and the plantar fascia. Sci Rep. 2020;10:14523. 32884015 10.1038/s41598-020-71316-z PMC7471908

[48] Hagen B Armstrong-Esther C Sandilands M. On a happier note: validation of musical exercise for older persons in long-term care settings. Int J Nurs Stud. 2003;40:347–57. 12667511 10.1016/s0020-7489(02)00093-7

[49] Jung YJ Min S Kim KS. The effect of rhythmic exercise program on serum lipid, superoxide dismutase, catalase activity in the elderly women. J Korean Assoc Phys Educ Sport Girls Women. 2004;18:1–20.

[50] Oak JS Park WY. Effects of resistance training on fitness and equilibrium sensory function in old adults. Exercise Sci. 2004;13:101–12.

[51] Schnelle JF MacRae PG Giacobassi K MacRae HS Simmons SF Ouslander JG. Exercise with physically restrained nursing home residents: maximizing benefits of restraint reduction. J Am Geriatr Soc. 1996;44:507–12. 8617897 10.1111/j.1532-5415.1996.tb01434.x

[52] Pinckard K Baskin KK Stanford KI. Effects of exercise to improve cardiovascular health. Front Cardiovasc Med. 2019;6:69. 31214598 10.3389/fcvm.2019.00069 PMC6557987

[53] Yoshizawa M Maeda S Miyaki A. Effect of 12 weeks of moderate-intensity resistance training on arterial stiffness: a randomised controlled trial in women aged 32-59 years. Br J Sports Med. 2009;43:615–8. 18927168 10.1136/bjsm.2008.052126

[54] Albaladejo R Asmar MS Benetos A. Association between 24-hour ambulatory heart rate and arterial stiffness. J Hum Hypertens. 2000;14:137–41. 10723121 10.1038/sj.jhh.1000961

[55] Bassiouny HS Zarins CK Kadowaki MH Glagov S. Hemodynamic stress and experimental aortoiliac atherosclerosis. J Vasc Surg. 1994;19:426–34. 8126855 10.1016/s0741-5214(94)70069-9

[56] Mackey RH Sutton-Tyrrell K Vaitkevicius PV. Correlates of aortic stiffness in elderly individuals: a subgroup of the Cardiovascular Health Study. Am J Hypertens. 2002;15(1 Pt 1):16–23. 11824854 10.1016/s0895-7061(01)02228-2

[57] Lo HM Yeh CY Chang SC Sung HC Smith GD. A Tai Chi exercise programme improved exercise behaviour and reduced blood pressure in outpatients with hypertension. Int J Nurs Pract. 2012;18:545–51. 23181955 10.1111/ijn.12006

[58] Chandler MP DiCarlo SE. Acute exercise and gender alter cardiac autonomic tonus differently in hypertensive and normotensive rats. Am J Physiol. 1998;274:R510–6. 9486311 10.1152/ajpregu.1998.274.2.R510

[59] Jun JK Kim SH Jeon BH Kim IK. The effects of α-, β-adrenergic receptor blocker and endurance exercise on hypertension and aerobic capacity in spontaneously hypertensive rats. Exercise Sci. 1998;7:53–69.

[60] Sesso HD Paffenbarger RS Jr Lee IM. Physical activity and coronary heart disease in men: the Harvard alumni health study. Circulation. 2000;102:975–80. 10961960 10.1161/01.cir.102.9.975

[61] Tordi N Colin E Mourot L Bouhaddi M Regnard J Laurant P. Effects of resuming endurance training on arterial stiffness and nitric oxide production during exercise in elite cyclists. Appl Physiol Nutr Metab. 2006;31:244–9. 16770351 10.1139/h05-033

